# The gut-joint axis in gout: microbial outer membrane vesicles and m^6^A-mediated metabolic-epigenetic coupling from acute flare to chronicity

**DOI:** 10.3389/fimmu.2026.1854522

**Published:** 2026-06-08

**Authors:** Yiyang Qi, Jixiang Bai, Dewei Liu, Shuhui Wang

**Affiliations:** 1 Mudanjiang Medical University, Mudanjiang, China; 2 Hongqi Hospital Affiliated with Mudanjiang Medical University, Mudanjiang, China

**Keywords:** epigenetic memory, gout, gut-joint axis, histone lactylation, immunometabolism, m^6^A methylation, outer membrane vesicles (OMVs), systemic intervention

## Abstract

Gout is increasingly recognized as a systemic metabolic disorder driven by the “gut-joint axis” rather than a purely localized joint disease. However, the exact mechanisms by which intestinal dysfunction causes persistent joint inflammation remain unclear. This review proposes a novel “Two-Hit” theoretical framework mediated by bacterial outer membrane vesicles (OMVs) and N6-methyladenosine (m^6^A) epigenetic modifications. We hypothesize that the high uric acid environment in the gut exerts a metabolic stress on specific bacteria, driving the release of highly pathogenic OMVs. Following intestinal barrier damage, these OMVs, working synergistically with intestinal-derived lipopolysaccharide (LPS), act as cross-organ messengers to deliver a “two-hit” strike to the joint. First, they prime synovial macrophages (SMs) by upregulating m^6^A methylation (via the methyltransferase-like 3 enzyme, METTL3), creating a pro-inflammatory epigenetic memory. Second, they induce metabolic reprogramming, characterized by enhanced glycolysis and local acidification in synovial fibroblasts, which physically forces the crystallization of uric acid. Based on this theoretical model, we evaluate emerging therapeutic strategies. These include stabilizing bacterial membranes to block OMV release, using biomimetic nanotechnology to intercept circulating vesicles, and targeting m^6^A enzymes to erase inflammatory memory. Ultimately, this hypothesis suggests a potential framework to conceptually shift gout management from symptom relief toward source-to-epigenetic precision interventions, while highlighting the necessary directions for future experimental validation.

## 1 Introduction

Gout, a prototypical metabolic autoinflammatory disease characterized by monosodium urate (MSU) crystal-induced inflammation, has emerged as a burgeoning global health challenge with a distinct trend of rejuvenation. Driven by evolving dietary and lifestyle factors, the prevalence of gout is escalating rapidly, particularly among younger populations (ages 15–44), with projections suggesting the affected population will surpass 9.2 million by 2046 (1). Despite the ubiquity of urate-lowering and anti-inflammatory interventions, many patients remain refractory to treatment, suffering from persistent relapses and drug-related toxicities (2). This clinical reality has driven a transition from the narrow “joint-centric” view to a comprehensive systemic inflammatory framework (3, 4). Within this context, there is an urgent need to elucidate how extra-articular compartments contribute to and orchestrate the localized inflammatory storms that define gout flares.

In recent years, the burgeoning concept of the “gut-joint axis” has propelled gout research into a new paradigm of inter-organ crosstalk (5). Numerous metagenomic evidence has established that the intestinal microecology in gout patients is characterized by a significant impoverishment of microbial diversity and profound structural dysbiosis. Notably, the systemic depletion of butyrate-producing taxa—most prominently *Faecalibacterium prausnitzii*—alongside the aberrant expansion of Gram-negative pathogens such as Prevotella, serves as the microecological cornerstone of disease onset (6, 7). However, most extant literature remains largely descriptive, focusing on correlational analyses between microbial signatures and clinical phenotypes. A systematic mechanistic explanation for how gut-derived pathogenic signals navigate complex anatomical barriers to achieve precise delivery into the distal synovial space remains elusive. While the traditional “leaky gut” model accounts for the passive translocation of endotoxins, the inherent instability and rapid neutralization of free lipopolysaccharide (LPS) in systemic circulation suggest it is unlikely to be the primary mediator of such long-range inter-organ communication.

The elucidation of this knowledge gap has been facilitated by burgeoning research into OMVs. As nanoscale vehicles actively shed by Gram-negative bacteria, OMVs—shielded by their lipid bilayer membranes—ensure the stable sequestration and long-range transport of LPS, virulence factors, and bacterial nucleic acids, thereby orchestrating high-efficiency inflammatory signaling (8). Synthesizing recent evidence regarding BEV-mediated pathogenicity in arthritis models (9), we hypothesize that OMVs serve as the pivotal conduits for the remote delivery of gut-derived inflammatory signals to the joints. A more profound breakthrough involves the capacity of these gut-derived vehicles to not only trigger immune activation but also fundamentally reprogram the metabolic landscape of synovial cells via epigenetic modifications—most notably N6-methyladenosine (m^6^A). This metabolic-epigenetic coupling is considered a key turning point in the progression of gout from acute flare-ups to a chronic, refractory state (10).

Given the exponential growth of research in this field, there is a pressing need for a systematic integration of the complete pathological trajectory comprising “gut dysbiosis—OMV transport—epigenetic remodeling”. Herein, we propose a novel conceptual model for gout pathogenesis: the “OMVs-m^6^Amediated ‘Two-Hit’ hypothesis within the gut-joint axis.” We hypothesize that under the hyperuricemia (HUA) microenvironment characteristic of gout, highly pathogenic OMVs secreted by specific gut microbiota (e.g., *Prevotella* species) synergize with other gut-derived LPS to collectively target fd macrophages within the articular synovium. On the one hand, immunogenic gut-derived LPS upregulates m^6^A methylation levels in macrophages, epigenetically amplifying inflammatory precursors (establishing Signal 1). On the other hand, OMVs induce aerobic glycolysis in FLS, causing local acidification that physically drives the precipitation of MSU crystals (establishing Signal 2). This review aims to systematically elucidate this hypothesis and evaluate potential therapeutic interventions targeting this axis. It is crucial to emphasize that while the individual components of this axis—such as gut dysbiosis in hyperuricemia and the role of m^6^A in macrophage polarization—are established mechanisms in recent literature, their continuous integration into a gout-specific “gut-joint” signaling cascade remains a proposed hypothesis that warrants future experimental validation. Ultimately, we hope to provide a conceptual foundation for precision medicine and the reversal of disease progression in gout.

## 2 Intestinal microecological remodeling in gout

### 2.1 Ecological impoverishment of gut microbial diversity

The species diversity of the gut microbiota is a key cornerstone for maintaining the homeostasis of the microecosystem and host health. Clinical evidence consistently shows that diversity indices such as Shannon, Simpson, and Chao1—which reflect community richness and evenness—are significantly reduced in patients’ intestines (11–13). This reduction in diversity often signifies that the intestinal microbiome has lost its original balance and stability. Due to the lack of competition and suppression from abundant beneficial bacteria, the gut’s biological barrier function is weakened, thereby creating conditions conducive to the growth and proliferation of potential pathogenic bacteria.

### 2.2 Depletion of core functional bacterial genera and the reconfiguration of pro-inflammatory taxa

**Figure 1:**
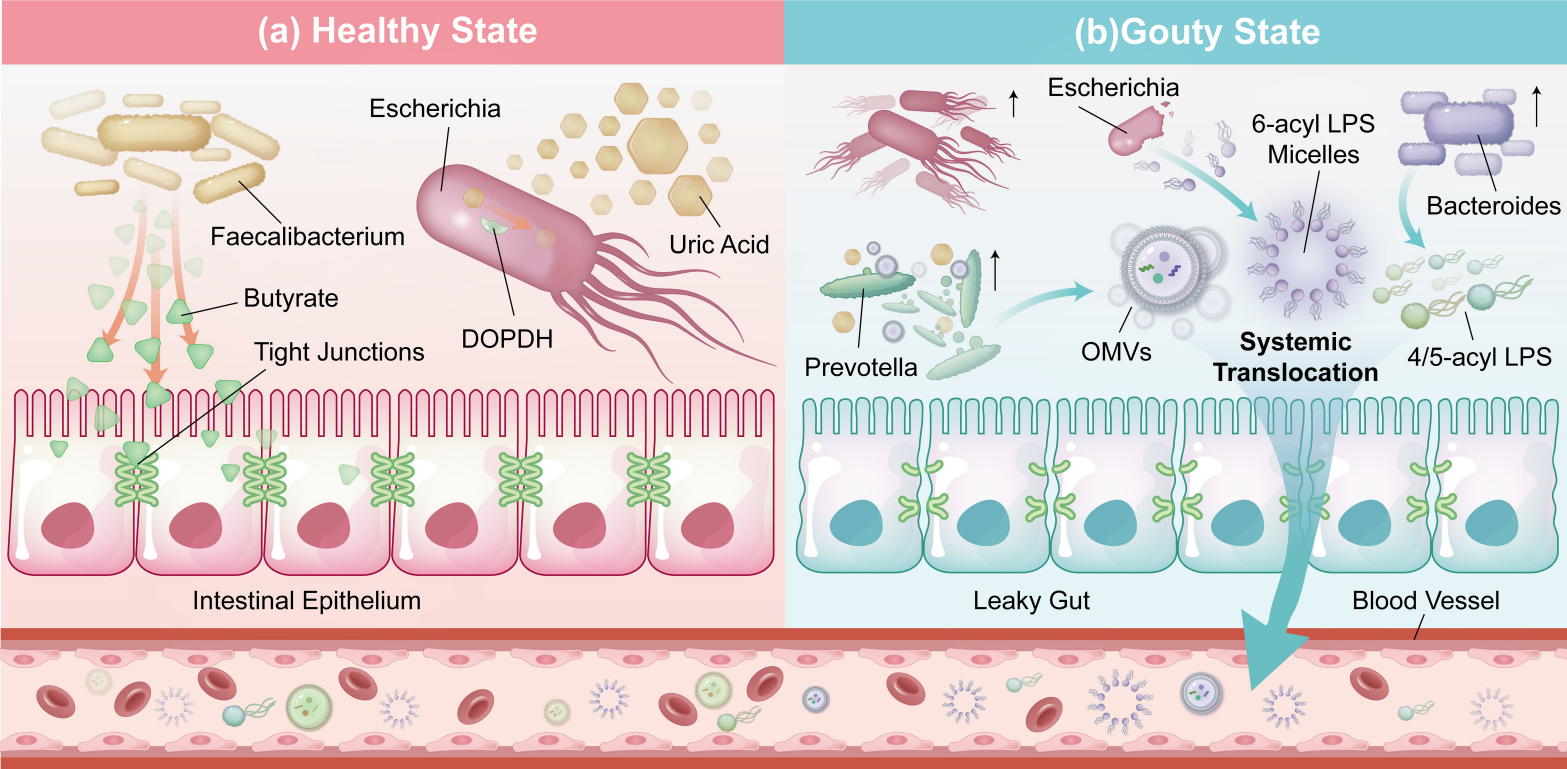
Microecological remodeling and the generation of pathogenic carriers along the gut-joint axis in gout. **(a)** Healthy State: Under homeostatic conditions, the intestinal epithelial barrier is structurally intact, maintained by functional tight junctions. Symbiotic bacteria, represented by *Faecalibacterium*, actively secrete short-chain fatty acids such as butyrate (indicated by green triangles), which reinforce barrier integrity. Concurrently, commensal strains like *Escherichia* contribute to maintaining purine homeostasis by expressing selenium-dependent 2,8-dioxopurine dehydrogenase (DOPDH) to degrade luminal uric acid (yellow hexagons). **(b)** Gouty State: Driven by the hyperuricemic (HUA) microenvironment and related dietary stressors, the gut microecology undergoes profound dysbiosis and structural collapse (“Leaky Gut”). The depletion of butyrate-producing *Faecalibacterium* leads to the downregulation of tight junction proteins. Consequently, opportunistic pathogens expand their metabolic niches and generate a massive payload of pathogenic carriers: (1) Due to high local oxidative stress and urate load, *Escherichia* undergoes massive bacterial lysis, explosively releasing highly immunogenic 6-acyl lipopolysaccharide (LPS), which spontaneously assembles into LPS micelles. (2) *Prevotella* aberrantly expands and actively secretes nanoscale outer membrane vesicles (OMVs) containing concentrated pathogen-associated molecular patterns (PAMPs). (3) *Bacteroides* proliferates and sheds massive amounts of 4/5-acyl LPS. Ultimately, the compromised intestinal barrier facilitates the systemic translocation of these highly pathogenic carriers (OMVs and LPS micelles) into the bloodstream, establishing the prerequisite for cross-organ “gut-joint” inflammatory signaling.

Current studies indicate that HUA and its progression to gout are driven by three core factors: high-purine diets, high-fructose intake, and genetic susceptibility (14, 15). These factors induce characteristic microbiota dysbiosis by remodeling the gut microenvironment. Excessive intake of exogenous purines provides a significant metabolic niche for purine-degrading bacteria (such as *Escherichia and Bacteroides*) (16). Because hominids lost the uricase gene during evolution (17), the broadly distributed bacterial urate oxidase gene cluster Hypoxanthine utilization (*hpx*) and the 2,8-dihydroxyadenine (2,8-DOP) degradation pathway in the gut have become crucial compensatory mechanisms for maintaining host purine homeostasis (18). However, the metabolic byproducts (e.g., endotoxins) accompanying this compensatory bacterial expansion disrupt intestinal mucosal homeostasis, thereby perpetuating the dysbiotic state. Concurrently, dietary fructose metabolism significantly downregulates intestinal tight junction proteins such as Zonula occludens-1 (ZO-1)and Occludin, directly impairing gut barrier integrity and inducing a “leaky gut” state (19). This increased permeability not only restricts the survival space of beneficial anti-inflammatory bacteria (e.g., *Faecalibacterium*), leading to their depletion, but also facilitates the systemic translocation of OMVs produced by pathogenic bacteria into the bloodstream. Consequently, these OMVs act as critical pathogenic carriers linking gut dysbiosis to systemic inflammation. Furthermore, genome-wide Mendelian randomization studies have confirmed that host genetic factors directly determine the initial abundance levels of key taxa, such as *Prevotella* (20). Additionally, functional impairment of urate transporters (e.g., ATP-binding cassette sub-family G member 2) due to genetic defects exacerbates the burden of intestinal urate excretion and alters the luminal pH and chemical gradients (21), which fundamentally establishes the ecological baseline for microbiota dysbiosis under disease conditions. Based on the microecological remodeling driven by these multidimensional factors, the following sections will detail the evolutionary characteristics of four core bacterial taxa and their specific pathogenic roles in the progression of gout (**Table 1**). Concurrently, the overarching morphological transition from a homeostatic intestinal barrier to a dysbiotic “leaky gut,” alongside the massive generation and systemic translocation of pathogenic carriers (LPS micelles and OMVs), is visually summarized in **Figure 1**.

**Table 1:** Characteristics and pathogenic mechanisms of core gut microbiota in gout.

Genus/Species	Trend	Key pathogenic/Mechanisms	Impact	Ref.
*Faecalibacterium (F. prausnitzii)*	↓↓Depletion	Molecular Features: Obligate anaerobe; suppresses NF-*κ*B; promotes TJP (ZO-1, Occludin) expression.	Structural depletion leads to “Leaky Gut”; systemic translocation of PAMPs and OMVs.	(22, 23)
		Butyrate (SCFAs), MAM (Anti-inflammatory Molecule)	Loss of immune buffering; systemic metabolic endotoxemia; reduced IL-10 levels.	
*Bacteroides (B. caccae, B. fragilis, B.* *xylanisolvens)*	↑↑Expansion	Molecular Features: Encodes urate oxidase cluster to utilize gut urate; source of low-immunogenicity LPS.	Niche expansion driven by high purine/urate load; displacement of butyrate producers.	(24, 25; 26)
		4/5-acylated LPS, hpx (Urate Oxidase Gene Cluster)	Systemic metabolic endotoxemia: potential inhibition of ABCG2 (urate transporter) via metabolic remodeling.	
*Prevotella (P. copri/S. copri)*	↑↑Expansion	Molecular Features: Secretes antigenic nanoscale lipid bilayer OMVs; potent degrader of mucin glycoproteins.	OMVs bypass physiological barriers; target distal synovial tissue via systemic circulation.	(20, 27–29)
		OMVs	Gut-Joint Axis cross-organ pathogenesis; NLRP3 activation; epigenetic modification of urate targets.	
*Escherichia (E. Coli)*	↑↑ High Turnover	Molecular Features: Encodes selenium-dependent DOPDH; coupled 2,8-DOP degradation with SCFA synthesis.	Mass lysis under urate stress; explosive release of potent hexacylated Lipid A via envelope dissolution.	(20, 30; 31)
		6-acylated LPS (Potent), DOPDH (2,8-dioxopurine pathway)	Severe structural barrier collapse; systemic inflammatory priming (TLR4/MD-2); renalurate excretion damage.	

Arrows (↑↑*/*↓↓) indicate significant enrichment or depletion in gout patients compared to healthy controls based on current metagenomic consensus. High turnover refers to intensified bacterial lysis and envelope dissolution observed under high uric acid microenvironments, leading to explosive endotoxin release.

UA, Uric Acid; OMVs, Outer Membrane Vesicles; LPS, Lipopolysaccharides; TJP, Tight Junction Proteins; MAM, Microbial Anti-inflammatory Molecule; hpx, Urate Oxidase Gene Cluster; DOPDH, Dioxopurine Dehydrogenase; SCFAs, Short-Chain Fatty Acids; TLR4, Toll-like Receptor 4; NLRP3, NLR family pyrin domain containing 3. This table summarizes the evolutionary trends and molecular pathways of key bacterial genera involved in the gut-joint axis during the progression of gout. The depletion of anti-inflammatory butyrate-producers (*Faecalibacterium*) and the aberrant accumulation or high turnover of opportunistic pathogens (Bacteroides, *Prevotella*, and *Escherichia*) collectively contribute to the erosion of the intestinal epithelial barrier (“leaky gut”) and the subsequent systemic translocation of metabolic endotoxins (LPS and OMVs).

#### 2.2.1 Genus *Faecalibacterium*

*Faecalibacterium*, particularly *F. prausnitzii*, constitutes one of the most prevalent obligate anaerobes within the human intestinal tract. As a hallmark butyrate-producing taxon, *F. prausnitzii* manifests as systemic exhaustion in the course of gout (22, 23). The resulting butyrate deficiency downregulates the expression of tight junction proteins (TJPs), directly increasing intestinal epithelial permeability and facilitating a ‘leaky gut’ phenotype. Anatomically, this “leaky gut” architecture not only facilitates the systemic translocation of free LPS and other Pathogen-associated molecular patterns (PAMPs) but also affords a physical conduit for OMVs shed by pathogens such as *Prevotella* to bypass physiological barriers, infiltrate the lamina propria, and gain access to the systemic circulation (32). Furthermore, *F. prausnitzii* exhibits profound anti-inflammatory properties, notably through the secretion of the microbial anti-inflammatory molecule (MAM). This protein suppresses the production of pro-inflammatory cytokines by inhibiting the nuclear factor kappa-light-chain-enhancer of activated B cells (NF-*κ*B) signaling pathway (33). A decline in the abundance of this bacterium deprives the gut of a vital immunosuppressive buffer, promoting a shift toward a pro-inflammatory microenvironment characterized by reduced IL-10 expression. This localized immune compromise, coupled with systemic metabolic endotoxemia, facilitates the evasion of host immune clearance by virulence factors, ultimately triggering the “gut-joint axis” pathogenic cascade.

#### 2.2.2 *Bacteroides* and *Prevotella*

The genus *Bacteroides* constitutes one of the most predominant taxa in the human gut and is primarily responsible for the degradation of complex carbohydrates (34). In gout patients, this genus harbors the widely distributed urate oxidase gene cluster (hpx) (17), enabling it to efficiently metabolize the abundant uric acid and purine substrates that enter the intestinal lumen via compensatory intestinal excretion (which accounts for approximately one-third of total body urate elimination), thereby achieving ecological niche expansion. Characterized by the enrichment of specific species, including *B. caccae*, *B. fragilis*, and *B. xylanisolvens* (20). *Bacteroides* serves as the primary molecular donor of LPS within the intestinal lumen. In contrast to the highly immunogenic hexa-acylated LPS characteristic of *Escherichia coli*, LPS derived from *Bacteroides* typically features a tetra-acylated or penta-acylated structure (24). Within the pathological context of *F. prausnitzii* depletion and the subsequent compromise of the intestinal barrier, *Bacteroides* exploits its numerical advantage to translocate into the bloodstream on a large scale, thereby inducing systemic metabolic endotoxemia (25, 26). Furthermore, the expansion of *Bacteroides* often occurs in tandem with a further reduction in butyrate-producing commensals, fostering a vicious cycle of progressively declining anti-inflammatory capacity (11). The enrichment of specific species, such as *B. caccae*, is closely associated with extensive remodeling of the host intestinal metabolome. The resulting disturbances in secondary bile acid and amino acid metabolism may indirectly impair the excretory function of key urate efflux pumps—specifically ATP-binding cassette super-family G member 2 (ABCG2)—while simultaneously altering the physicochemical properties of the intestinal lumen, ultimately leading to a compensatory elevation in serum uric acid levels (7, 35, 36).

The role of the genus *Prevotella* in gout and HUA exhibits a complex bidirectional nature, with its pathogenic or protective effects highly dependent on specific strain types and host genetic backgrounds (37). Notably, under the pathological context of gout, the aberrant expansion of *Prevotella copri (P. copri)* is intimately tied to the host’s HUA metabolic state and high-purine dietary habits (38). This unique metabolic milieu confers a selective advantage upon *P. copri*, enabling it to aggressively outcompete beneficial, butyrate-producing commensals. Ecologically, the overgrowth of *P. copri* imposes a profound pathogenic burden within the intestinal lumen by acting as a potent mucosal barrier disruptor. A high abundance of *P. copri* correlates with the excessive degradation of mucin glycoproteins and the downregulation of tight junction proteins, actively facilitating a “leaky gut” architecture. Furthermore, *P. copri* is significantly enriched in patients with both gout and rheumatoid arthritis (RA), suggesting it may represent a shared microecological signature across these inflammatory arthropathies (20, 27). In both disease models, this pathobiont plays a critical role in maintaining a high background of systemic inflammation. By elevating circulating levels of LPS and other bacterial products effectively lowers the threshold for inflammatory flares in the joints. In the wellestablished context of RA, it has been robustly demonstrated that P. copri interacts with the host by secreting OMVs carrying antigenic proteins (39). Building upon this mechanistic paradigm, we deduce that in gout, *P. copri* similarly exploits these nanoscale vehicles to bypass physiological barriers. Therefore, we hypothesize that under the HUA background, enriched *P. copri* actively generates OMVs that breach the hyperpermeable intestinal mucosa, systemically traffic through the circulation, and distally enrich within synovial tissues.

#### 2.2.3 Genus *Escherichia*

Metagenomic analysis has confirmed that *Escherichia coli* (*E. coli)* exhibits a characteristic increase in abundance in the intestines of patients with gout and HUA (20). Recent breakthroughs have redefined the role of *E. coli* in intestinal urate metabolism, identifying a unique anaerobic 2,8-DOP pathway (16, 40). Research indicates that *E. coli* expresses a selenium-dependent 2,8-dioxopurine dehydrogenase (DOPDH), which utilizes uric acid—excreted from the systemic circulation into the gut lumen—as a carbon source. This degradation process is tightly coupled with the biosynthesis of short-chain fatty acids (SCFAs) (18). Concurrently, the overgrowth of *E. coli* is accompanied by an exceptionally high bacterial turnover rate. Current research indicates that under the localized high oxidative stress within the gut, the massive shedding of free hexa-acylated LPS—a structural component of the outer membrane—by *E. coli* relies primarily on bacterial cell death and envelope lysis (30, 31), rather than active secretion. Beyond triggering systemic inflammation, this profound metabolic endotoxemia also exacerbates “underexcretion-type” HUA by impairing renal function. Compared to the weakly immunogenic, deacetylated LPS derived from *Bacteroides*, the hexa-acylated Lipid A released during *E. coli* lysis serves as a potent agonist for the Toll-like receptor 4 (TLR4)/myeloid differentiation factor 2 (MD-2) complex (17, 30). This potent endotoxin, concentrated and exposed in micellar form (31), drives the collapse of the intestinal barrier by significantly downregulating tight junction proteins, such as occludin (41). Critically, this highly inflammatory LPS exhibits a significant synergistic priming effect with the high-abundance *Bacteroides* LPS (42). By lowering the threshold for immune activation, they jointly catalyze the onset of systemic metabolic endotoxemia.

## 3 Mechanisms of cross-organ communication along the gut-joint axis

Compromised intestinal barrier integrity facilitates the systemic translocation of enteric PAMPs. These macromolecular mediators infiltrate the bloodstream and become distally localized within the synovial environment, serving as a critical immunological pre-priming signal.

### 3.1 Hematogenous spread of enteric pathogens

Disruption of the intestinal physical barrier enables luminal PAMPs to translocate through the lamina propria and gain access to systemic circulation. Given the inherent hydrophobicity of Lipid A (the bioactive anchor of LPS) these molecules are unable to persist as monomers within the aqueous environment of the bloodstream. Consequently, the physical architecture and dissemination kinetics of blood-borne LPS are governed by the metabolic state of the source microbiota, resulting in two distinct transport modalities.

#### 3.1.1 Systemic translocation of free LPS via host EV capture

In the intestines of gout patients, resident flora such as *E. coli* undergo massive lysis induced by oxidative stress or immune clearance in the HUA microenvironment (31). This physical disruption of the cell wall releases highly immunogenic hexacylated LPS, which spontaneously assembles into linear or spherical micelles. Traditionally, it was understood that lipopolysaccharide-binding protein (LBP) extracts LPS monomers from these micelles and transfers them to high/low-density lipoprotein (HDL/LDL) particles—a pathway primarily destined for hepatic clearance and detoxification (43). However, recent breakthroughs in systemic intracellular surveillance have fundamentally redefined the fate of circulating free LPS. It has been compellingly demonstrated that host-derived extracellular vesicles (EVs) (44), which are highly abundant in the bloodstream, possess a remarkable intrinsic capacity to capture blood-borne LPS via direct lipid bilayer-Lipid A interactions (45). Building upon this established biological fact, we postulate a critical paradigm shift in gout pathogenesis: under the condition of a “leaky gut”, the massive paracellular influx of micellar LPS overwhelms the conventional HDL/LDL detoxification capacity. Consequently, the circulating host EVs act as a systemic sponge, rapidly adsorbing the spilled intestinal LPS. Crucially, unlike lipoprotein-bound LPS, EV-bound LPS evades hepatic clearance, acquiring a highly stable and sheltered transit to distant compartments. Nevertheless, a critical scientific caveat must be rigorously acknowledged. While the host EV-LPS coupling paradigm has been validated in broader endotoxemia models, direct empirical evidence identifying these specific LPS-laden host EVs within the circulation of gout or HUA patients is currently lacking. Consequently, high-resolution *in vivo* EV tracking and clinical proteomic validation are strictly required to elevate this theoretical “gut-joint” relay from a conceptual hypothesis to a definitive pathological mechanism.

#### 3.1.2 Intestinal-derived OMVs

When investigating the cross-organ transport of pathogenic signals, the physical morphology of the carrier dictates its survival rate within the systemic circulation. Current literature in fundamental microbiology and immunology has established that, in contrast to the free LPS micelles shed during the passive lysis of *E. coli*, pathogenic commensals such as *Prevotella* actively secrete nanoscale OMVs via outer membrane blebbing, driven by active metabolic processes. OMVs possess an intact lipid bilayer structure, a conformation that shields the encapsulated specific LPS, metabolic enzymes, and bacterial nucleic acids from degradation by serum enzymes (46). Their robust spherical spatial conformation confers a structural resistance far superior to that of free LPS micelles against the extracting activities of lipopolysaccharidebinding protein (LBP) or high-density lipoproteins (HDL) in the blood. Consequently, this ensures that gut-derived pathogen-associated molecular patterns (PAMPs) maintain an exceptionally high viability within the circulation. Leveraging their nanoscale dimensions, they can traverse interendothelial gaps to achieve targeted delivery to distal synovial tissues (47).

There is evidence from basic immunology indicating that, upon reaching the synovial microenvironment, intestinal-derived OMVs interact physically with local host cells (48). Because OMVs act as carriers of high concentrations of PAMPs, specific ligands enriched on their surface (such as LPS and the outer membrane protein A, OmpA) can be effectively recognized and bound by pattern recognition receptors on the surface of synovial FLS and synovial macrophages (SMs) (49). In the synovial microenvironment, the interaction between OMVs and host cells depends on specific receptor recognition and active uptake. Studies have shown that, unlike the passive diffusion of free monomeric molecules, the uptake of OMVs by host cells is highly dependent on active endocytosis pathways. OMVs are efficiently internalized primarily through mechanisms such as lipid raft-mediated, clathrin-mediated, or macropinocytosis (47). Furthermore, existing studies have clearly demonstrated that synovial cells also possess the ability to actively internalize pathogen outer membrane vesicles and trigger downstream inflammatory responses (50). Recent research reveals that LPS in this vesicle-bound configuration gains entry to target cells via a CD14-dependent endocytic pathway, subsequently bypassing early endosomes to release concentrated LPS into the host cell cytoplasm (45, 51). Although the metabolically adapted endocytic subtypes involved in the uptake of *Prevotella* outer membrane vesicles by synovial FLS and SMs in gout require further confirmation, this vesicle-based general endocytic mechanism confers significant biological advantages to the pathogen’s signaling.

### 3.2 Intestinal-derived PAMPs-driven microenvironmental remodeling and m^6^A-mediated metabolic reprogramming

While P. copri and its OMVs are implicated in the pathogenesis of both rheumatoid arthritis (RA) and gout, their pathogenic mechanisms fundamentally differ. In RA, they are associated with antigen-presented autoimmunity driven by genetic susceptibilities (e.g., HLA-DRB1), whereas in gout, they are linked to crystal-driven autoinflammation. Accordingly, we postulate that the disease-specificity of gout is primarily determined by the baseline microenvironmental state of the target synovial joint, rather than an exclusive structural tropism of the vesicles themselves. Pathophysiological evidence has already been established that HUA and its attendant physicochemical processes are absolute prerequisites for urate crystallization (52). Clinically, gout preferentially afflicts distal appendicular joints, such as the first metatarsophalangeal joint. Due to chronic biomechanical stress or subclinical pre-existing osteoarthritis, the local synovial vasculature in these regions is often in a state of pre-existing micro-vascular compromise, essentially presenting a ‘leaky joint’ phenotype. Concurrently, driven by systemic uric acid overload, the local interstitial fluid hovers at the critical threshold of uric acid supersaturation. Therefore, we postulate that this dual structural and biochemical baseline abnormality serves as a natural vascular homing beacon, facilitating the targeted extravasation and accumulation of circulating gut-derived macromolecules specifically into these vulnerable joint spaces.

**Figure 2:**
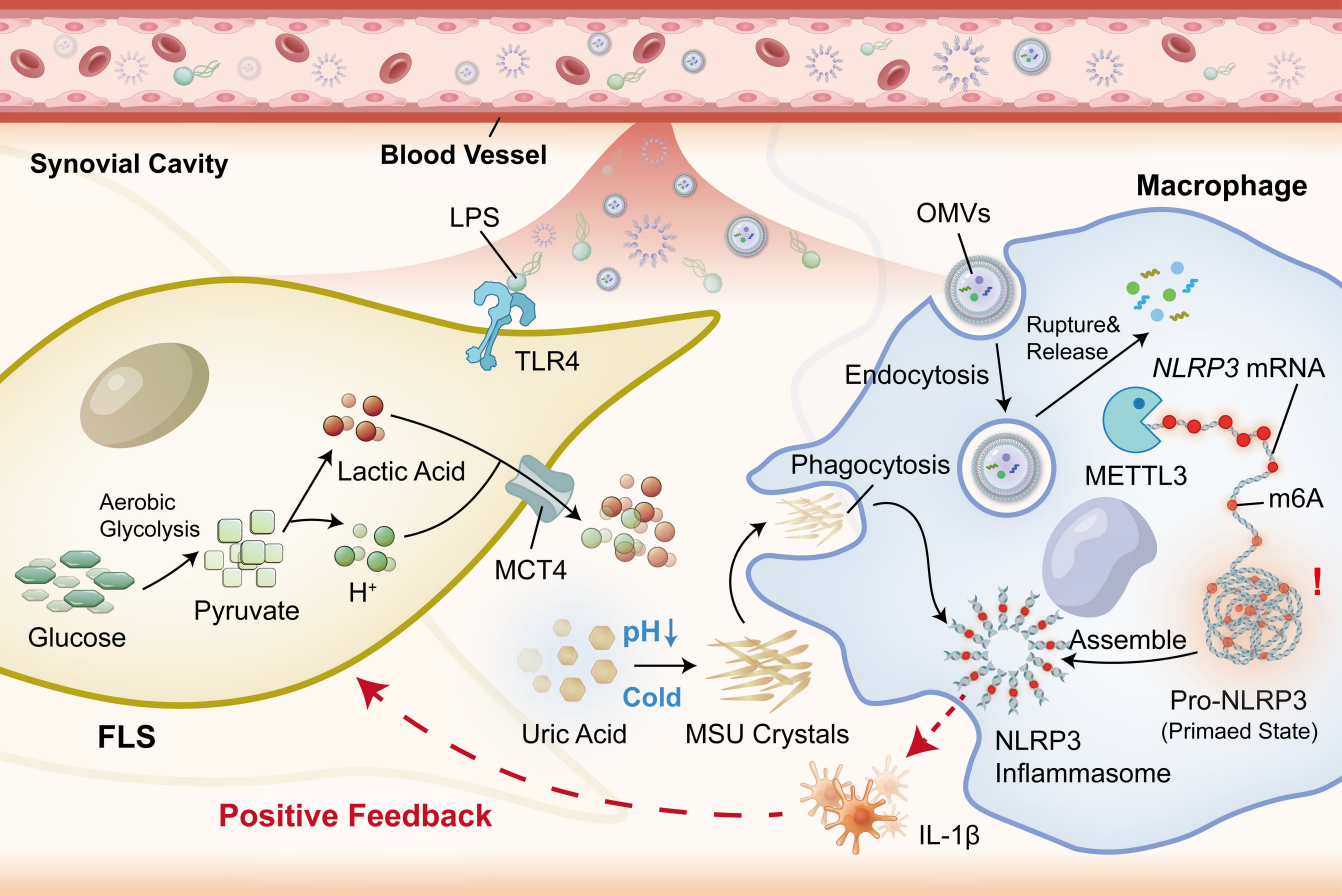
The “Two-Hit” metabolic-epigenetic cascade mediated by gut-derived carriers in the gouty synovium.

Recent evidence highlights m^6^A modification as a critical bridge amplifying inflammatory responses (53, 54). In classical acute organ injury models (55), pathogenassociated molecular patterns (PAMPs) act as potent upstream signals that significantly upregulate the expression of the m^6^A methyltransferase-like 3 enzyme (METTL3), thereby triggering a cascade-amplified inflammatory storm. Drawing upon this established “inflammation-m^6^A” positive feedback model, we propose a “Metabolic-Epigenetic Coupling” hypothesis driven by a coordinated “Two-Hit” mechanism within the gouty joint (**Figure 2**). On one hand, gut-derived LPS serves as a potent priming stimulus. By infiltrating the compromised synovium and hijacking the METTL3/m^6^A epigenetic machinery, LPS preamplifies and sequesters pro-inflammatory precursors within SMs at the post-transcriptional level, providing the requisite molecular foundation (Signal 1) (**Figure 2**, right panel). On the other hand, a substantial influx of microbial OMVs targets FLS, instigating profound metabolic reprogramming. The resulting extracellular efflux of lactate and protons leads to an overload of the local microenvironment, thermodynamically driving the *in situ* precipitation of MSU crystals (**Figure 2**, left panel). These crystals subsequently provide the essential physical trigger (Signal 2) to activate the epigenetically primed macrophages.

#### 3.2.1 SMs immune priming

Although existing research establishes that the acute flare of gouty arthritis is hallmarked by MSU crystal-induced NLRP3 activation, its initiation phase may depend largely on the pre-programming of the immunological background by LPS (56, 57). Although direct *in vivo* evidence linking m^6^A modifications specifically to gouty macrophages remains limited, extensive data from other acute organ injury models robustly demonstrate that LPS can act as a potent upstream stimulus to induce METTL3. Therefore, we hypothesize a similar epigenetic priming mechanism may occur in the gouty synovium. In this context, we draw upon models from the fields of macrophage polarization and epitranscriptomics. Frontier studies have demonstrated that LPS can act as a potent upstream stimulus to induce the high expression of the core m^6^A methyltransferase METTL3 within macrophages. The activation of METTL3 orchestrates the coordinated programming of the transcription and translation of NLRP3 and related pro-inflammatory factors as early as the nascent RNA stage (58). This not only significantly enhances target mRNA stability but also ensures the highly efficient expression of inflammatory proteins by coordinating transcription rates with translation initiation sites. While the LPS-induced METTL3 activation is a well-established mechanism in other acute injury models, its specific involvement in gouty arthritis currently lacks direct *in vivo* validation. Building upon these established facts, we propose a novel hypothesis: the early sensitization phase in gout might involve the infiltration of gut-derived LPS, which preferentially and potently activates resident SMs. By hijacking the aforementioned METTL3/m^6^A epigenetic machinery, this process enables SMs—even during the resting phase prior to crystal encounter—to stockpile an overabundance of NLRP3 inflammasome components and precursor cytokines (e.g., pro-IL-1*β*) at the post-transcriptional level, effectively establishing “Signal 1.” This ensures their capacity to mount a high-intensity inflammatory flare upon subsequent exposure to crystal stimulation.

#### 3.2.2 FLS microenvironment remodeling

The aforementioned mechanisms provide a novel perspective to address a core clinical paradox in gout: why do the majority of patients with asymptomatic HUA never experience an arthritis flare during their lifetime? We postulate that this hinges on whether the gut microenvironment has crossed a specific “stress threshold.” During the asymptomatic HUA phase, patients may only experience a low-level influx of generalized LPS into the bloodstream and enter the damaged synovial membrane, which is sufficient to maintain the immunologically primed state of SMs (i.e., remaining in a “standby” phase with only Signal 1). However, when the body encounters intense stimulation from external triggers (e.g., high-purine/high-fat diets, alcohol consumption, infections), the gut microecological homeostasis completely collapses, reaching the pathological stress threshold. At this juncture, pathogenic bacteria, represented by *Prevotella*, not only surge in abundance but actively secrete massive amounts of highly invasive OMVs into the circulation. It is precisely the massive influx of these threshold-breaking OMVs that constitutes the critical variable triggering “Signal 2.”

We draw upon the classical model of cellular metabolism established in the pathogenesis of RA. Specifically, FLS exhibit highly conserved features of metabolic reprogramming in response to stimulation by pathogen-associated molecular patterns (59). Intestinal-derived OMVs and their highly enriched LPS likely target and activate the TLR4 receptors on the surface of FLS. This activation stabilizes hypoxia-inducible factor-1*α* (HIF-1*α*), which subsequently suppresses mitochondrial oxidative phosphorylation in favor of intense aerobic glycolysis (i.e., the Warburg effect). This metabolic phenotypic switch leads to a massive intracellular accumulation of metabolic byproducts—lactate and protons (H+)—which are continuously effluxed into the extracellular synovial microenvironment via monocarboxylate transporters (MCTs) (60). Because the synovial cavity possesses a minute volume (approximately 1–2 mL) and acts as a semi-closed physical space (61), the effluxed H+ rapidly depletes the limited bicarbonate buffering capacity of the synovial fluid. When the local acidic load exceeds its compensatory threshold, the synovial fluid pH undergoes a significant downward pathological shift from its physiological baseline (approx. pH 7.4), typically plummeting to 7.0 or even lower during the peak of inflammation (62). Based on these dynamics, we postulate that the OMV-driven glycolytic acidification by FLS does not initiate uric acid accumulation *de novo*. Rather, it acts as the critical thermodynamic tipping point. The localized drop in pH, superimposed on the pre-existing state of urate supersaturation (as mentioned above) and the characteristically lower physical temperatures of distal joints, rapidly breaks the physicochemical equilibrium. This terminal metabolic collapse forces the already supersaturated urate to precipitously crystallize into MSU microcrystals on damaged cartilage surfaces, thereby supplying the ultimate physical trigger for the acute flare. Clinically, gout is highly predisposed to distal joints (e.g., the first metatarsophalangeal joint), where physiological temperatures are chronically maintained at lower levels (approximately 32°C) (63). These two factors—acidification and lower temperature—synergize to drastically compress the thermodynamic solubility of uric acid. This facilitates the precipitation of urate, leading to the formation of microcrystals on exposed type II collagen at damaged cartilage surfaces and within synovial plicae.

#### 3.2.3 m^6^A-mediated acute inflammation and epigenetic memory

The pathological progression of gout is not merely a sequence of discrete acute episodes but represents a continuous process governed by m^6^A methylation. During acute flares, METTL3-mediated m^6^A modifications significantly accelerate this inflammatory cascade. As mentioned earlier, METTL3 coordinates the transcription and translation of NLRP3 inflammasome during the nascent RNA stage. This epigenetic coordination enables synovial macrophages to produce high concentrations of pro-inflammatory cytokines, primarily IL-1*β*, upon stimulation by MSU crystals (58). IL-1*β* acts on adjacent FLS, driving the remodeling of glucose metabolism by upregulating their endogenous METTL3 activity. Specifically, METTL3-mediated m^6^A modification facilitates the initial enhancement of glycolysis by activating signaling pathways such as the HIF-1*α* axis (64). Relevant studies have corroborated that lactate, generated by high-intensity cellular glycolysis, acts not merely as a metabolic byproduct but as a crucial epigenetic substrate. Lactate can directly upregulate the expression and activity of target genes, such as METTL3, at the transcriptional level by inducing histone lactylation (65, 66), maintaining a sustained high level of lactate secretion. This also leads to increased stability of the NLRP3 inflammasome and accelerates the maturation of IL-1*β*. Furthermore, IL-1*β* can induce FLS and endothelial cells to subsequently secrete larger amounts of chemokines such as C-X-C motif chemokine ligand 1 (CXCL1) and CXCL8, which act in concert with the chemokines initially secreted by macrophages to guide the transendothelial migration of neutrophils (67). Early, scattered neutrophil extracellular traps (NETs) release granule proteins such as neutrophil elastase (NE), which directly process and activate extracellular pro-IL-1*β* through a non-canonical pathway. This mechanism establishes a potent pro-inflammatory positive feedback loop that dramatically amplifies the localized inflammatory response (60, 68, 69).

**Figure 3:**
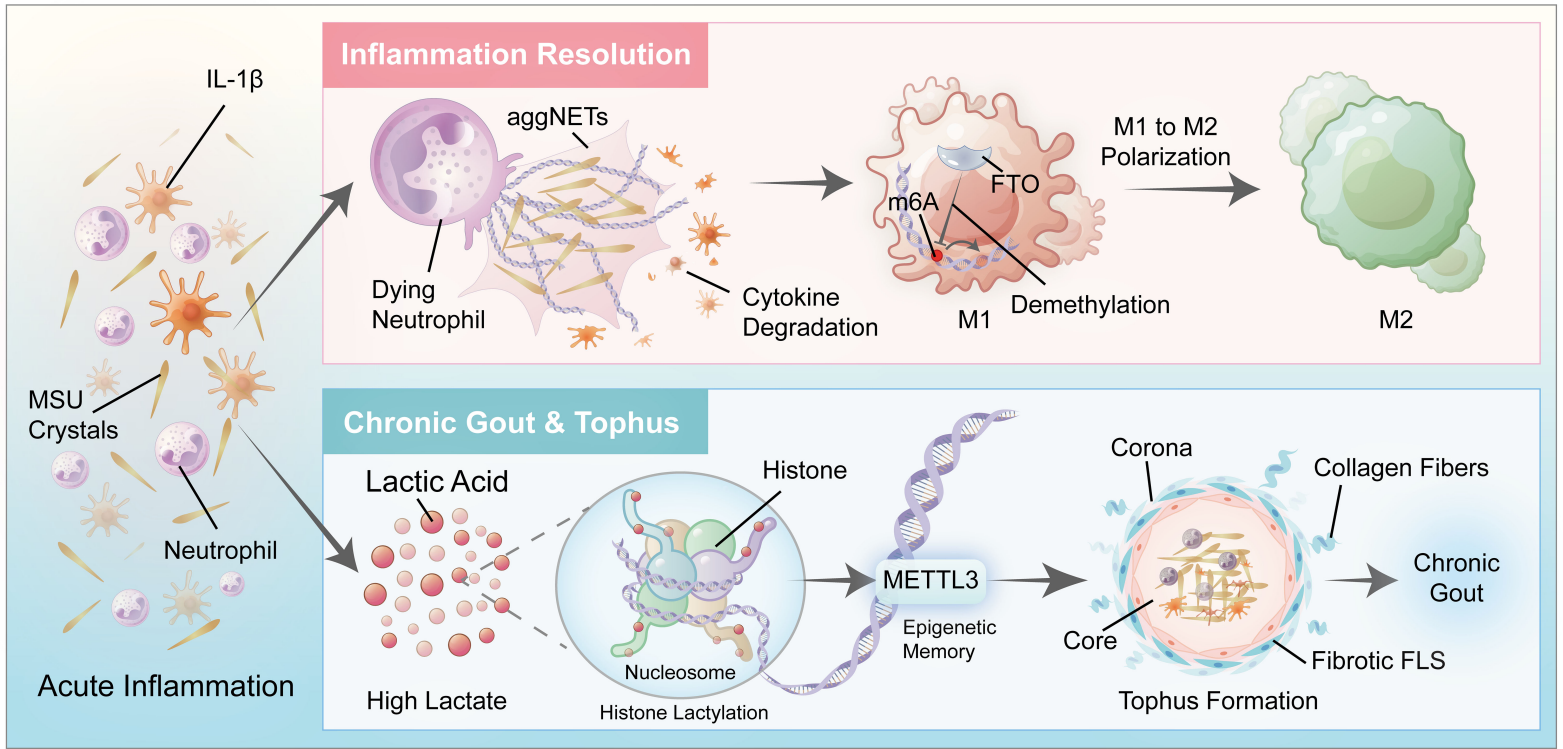
Divergent trajectories of acute gout flares: metabolic-epigenetic regulation from inflammation resolution to chronicity and tophus formation.

However, gouty inflammation is highly self-limiting. As neutrophils become highly concentrated within the joint cavity, scattered NETs gradually collapse and aggregate into high-density aggregated NETs (aggNETs) (70). This structural configuration not only isolates MSU crystals from continued stimulation through a physical encapsulation effect but also concentrates high levels of proteases on its surface to extensively degrade local cytokines and chemokines, thereby driving gout into a selfresolving phase (**Figure 3**, upper panel). Studies have confirmed that immune cells undergo metabolic reprogramming in response to local pathogen-derived signals and cytokines, and these metabolic changes significantly influence the induced immune response (71). In the pathological progression of gout, this metabolic remodeling represents a core factor determining the self-limiting nature of inflammation. Recent studies indicate that the resolution of gouty inflammation largely depends on the successful transition of macrophages from the pro-inflammatory M1 phenotype to the anti-inflammatory M2 phenotype. Within this process, targeted inhibition of the AHNAK nucleoprotein (AHNAK) effectively regulates the balance between glycolysis and fatty acid oxidation, which drives this phenotypic conversion and accelerates inflammation resolution (72). Although direct evidence regarding m^6^A methylation during the resolution phase of gout is limited, its role as a core mechanism regulating macrophage metabolic and functional polarization has been confirmed in other disease models. According to a 2024 study on diabetic nephropathy, the demethylase fat mass and obesity-associated protein (FTO) can precisely downregulate the intensity of aerobic glycolysis via the m^6^A/Npas2/HIF-1*α* axis, thereby inhibiting M1-type activation and inducing the M2-type reparative phenotype (73). Based on these findings, it is hypothesized that m^6^A modification may mediate cross-talk between epigenetic and metabolic remodeling through synergistic interactions with key proteins such as AHNAK, jointly determining the clinical course of gout.

Although the acute-phase chemotaxis and immune storm subside due to the formation of aggNETs and macrophage polarization, the bioenergetic state of the synovial tissue may not be fully restored. Recent studies suggest that the resolution phase following an acute gout flare may be a process in which epigenetic remodeling leaves a molecular-level immunological imprint (10). This immunological memory manifests as the persistent enrichment of histone modifications, such as H3K4me3, in the promoter regions of pro-inflammatory factors within the cellular genome, thereby maintaining cells in a long-term primed state (74). Direct evidence remains limited regarding whether metabolites accumulated during acute gout consolidate this immune state by inhibiting demethylases; however, this metabolicepigenetic coupling mechanism has been robustly confirmed in models of trained immunity (75). The accumulation of succinate and fumarate resulting from dysregulation of the tricarboxylic acid (TCA) cycle competitively inhibits the activity of *α*-ketoglutarate (*α*-KG)-dependent histone demethylases. This inhibition prevents the erasure of pro-inflammatory chromatin marks and locks immune cells in a heightened pro-inflammatory state. Such a mechanism explains why the metabolic state and immune microenvironment of synovial tissue in gout patients fail to return to homeostasis even after clinical remission of inflammation. Therefore, intervening in macrophage metabolism by targeting key metabolic nodes may not only induce the immediate resolution of inflammation but also break the inflammatory memory in synovial tissue. This is achieved by blocking feedback signals along the metabolic-epigenetic axis, thereby preventing the progression of gout to a chronic state. Concurrently, as the disease progresses, aggNETs generated during the acute, self-limiting phase exert dual roles. Beyond their anti-inflammatory effects, they physically serve as biological scaffolds for MSU crystal deposition (76). Studies indicate that FLS are recruited to these deposition sites and undergo a pro-fibrotic phenotypic transformation. Under conditions of sustained m^6^A-mediated metabolic remodeling and a locally acidified environment, these microscopic tophus-like structures recruit FLS to secrete a collagen matrix. These structures are eventually encapsulated by a fibrous capsule and evolve into clinically visible, irreversible tophi (77) (**Figure 3**, lower panel).

## 4 Therapeutic paradigm shift targeting the “gut-joint axis”

Conventional therapies for gout have been confined to localized urate-lowering and broad-spectrum antiinflammatory interventions, which fail to address the systemic root causes underlying recurrent disease flares. Based on the proposed “Dual Carrier-Two Hit” pathological model, future intervention strategies may pivot away from generalized immunosuppression toward precise spatiotemporal interception of specific nodes along this axis. This section evaluates current advancements and explores speculative intervention directions targeting this novel axis.

### 4.1 Targeted interventions modulating the gut microbiota

Precision interventions targeting the gut microbiota and its metabolites have evolved from merely reconstituting microbial abundance to the profound integration of metabolic pathway modulation and immune homeostasis restoration. Current clinical evidence demonstrates that interventions such as the consumption of electrolyzed alkaline water (EAW) can significantly enrich the abundance of butyrateproducing bacteria, including Faecalibacterium, thereby ameliorating the increased intestinal permeability induced by factors like high-fructose diets (78). Mechanistically, this strategy aims to intercept the transmucosal translocation of endotoxins (LPS) produced by pathogenic bacteria into the systemic circulation. By addressing the source, it holds the potential to alleviate LPS-mediated functional impairment of renal ABCG2 and glucose transporter 9 (GLUT9), thereby facilitating the restoration of urate excretion. Given the evolutionary loss of uricase in humans, emerging intervention strategies are attempting to harness the compensatory urate-degrading capabilities of the gut microbiota. Current precision interventions seek to exploit the widely distributed hpx gene cluster in *E. coli* and *Bacteroides* to activate the 2,8-DOP degradation pathway. Through molecular induction, these bacteria are stimulated to directly convert xanthine into 2,8-DOP within the intestinal lumen for ultimate degradation, thereby bypassing the host’s xanthine oxidase (XO) pathway. However, the overgrowth of wild-type *E. coli* is invariably accompanied by a markedly elevated risk of LPS release. To circumvent this, frontier research is dedicated to re-engineering *E. coli* into a “smart theranostic chassis.” Engineered *E. coli* developed via synthetic biology—serving as live biotherapeutic products (LBPs) (79)—can theoretically utilize urate-responsive promoters to achieve real-time sensing of luminal urate concentrations and specifically induce the expression of urate oxidase. This programmable genetic circuit retains the purine-scavenging potential while circumventing the risk of systemic endotoxemia triggered by the substrate-driven overgrowth of wild-type strains.

### 4.2 Intercepting the cross-organ transport and cellular uptake of intestinal-derived OMVs

The primary phase of intervention lies in reducing the secretory load of OMVs from intestinal pathogenic bacteria. Studies have corroborated that OMV release in Gram-negative bacteria is tightly orchestrated by envelope integrity, which involves key proteins that anchor the outer membrane to the peptidoglycan layer, such as the colicin-tolerant (Tol)-peptidoglycan-associated lipoprotein (Pal) system and lipoproteins (80). Accordingly, it is postulated that developing enhancers for these anchoring proteins, or inhibiting periplasmic proteases that induce outer membrane instability, could significantly fortify the bacterial cell wall architecture, thereby reducing their blebbing rate within the compromised luminal microenvironment. Furthermore, the dysregulation of lipid asymmetry transport systems (such as the VacJ/Yrb pathway, also known as the Mla pathway) leads to the aberrant accumulation of outer membrane phospholipids, triggering spontaneous vesiculation (81). Intervening in the activity of the VacJ/Yrb system via metabolic modulation or small-molecule drugs to maintain outer membrane lipid homeostasis is regarded as a potential strategy to precisely curtail the systemic delivery of highly virulent LPS carriers by gut pathogens.

Given that the surface of OMVs is highly enriched with host-recognizable ligands (such as OmpA and LPS), leveraging biomimetic nanotechnology to execute *in situ* sequestration within the blood circulation holds immense scientific value. Based on current advancements in biomaterials,”nano-decoys” cloaked in synovial cell membranes or red blood cell membranes can be employed to competitively bind circulating OMVs (82). Research has demonstrated that macrophage membrane-camouflaged nanovesicles can neutralize and clear various pro-inflammatory PAMPs, including OMVs, via surface receptors (such as TLR4), thereby exhibiting excellent targeted anti-inflammatory efficacy in arthritis models. Moreover, drawing an analogy to the breakthrough biomimetic strategies currently used in targeted therapies for rheumatoid arthritis, utilizing nanoparticles coated with the membranes of regulatory FLS can evade immune clearance and competitively intercept circulating pathogenic carriers by mimicking the host cell interface. This provides a novel conceptual framework for blocking the synovial colonization of gut-derived PAMPs in the field of gout (83). At the local synovial level, current cell biology studies have confirmed that the internalization of OMVs by host cells is highly dependent on lipid raft- and clathrin-mediated endocytosis (47, 84). Based on this, we postulate that the local administration of lipid raft-depleting agents (such as methyl-*β*-cyclodextrin, M*β*CD) or endocytosis inhibitors in preclinical models could physically disrupt the cytosolic translocation of OMVs into FLS and macrophages. This represents not only a potential therapeutic intervention but also a crucial experimental strategy to reverse-validate the hypothesis that “the intracellular release of pathogenic payloads by OMVs may act as a critical step for triggering the gouty cascade.”

### 4.3 Exploring interventions targeting synovial m^6^a modifications and the “metabolic-epigenetic” feedback axis

Disrupting the profound coupling between the epigenetic priming in macrophages (Signal 1) and the metabolic collapse in FLS (Signal 2) represents another speculative paradigm for seeking novel therapeutic avenues for gout.

Regarding the aberrant m^6^A methylation in SMs, it must be emphasized that current intervention strategies remain purely hypothetical for gout. Due to the current paucity of direct clinical or *in vivo* evidence in gout-specific models, these concepts are heavily extrapolated from other inflammatory paradigms. Drawing insights from other inflammatory disease models, histone lactylation is a recognized driving factor that upregulates METTL3 (the m^6^A “writer”) (85, 86). Consequently, developing metabolic inhibitors that target the generation of lactyl-CoA could theoretically attenuate the overexpression of METTL3. Concurrently, the judicious activation of m^6^A demethylases using smallmolecule compounds may facilitate the erasure of m^6^A tags from pro-inflammatory transcripts (e.g., NLRP3), thereby accelerating their degradation. For methyltransferases centered around METTL3, the intervention paradigm has already transitioned from laboratory-based gene knockouts (54) toward highly selective pharmacological inhibition (58). Recent studies have demonstrated that employing highly selective inhibitors to pharmacologically block METTL3 can fundamentally deplete the m^6^A abundance on the pro-inflammatory target NLRP3, hastening its mRNA decay. Potentially, activating the fat mass and obesity-associated protein (FTO) and AlkB homolog 5 (ALKBH5) -mediated demethylation process via metabolic interventions or small molecules (87, 88) holds promise for dynamically eliminating pre-established pro-inflammatory tags. Future studies utilizing *in vivo* gout models are imperative to validate the efficacy of this “metabolic-epigenetic” dual-blockade strategy in alleviating chronic inflammatory memory.

Concerning aerobic glycolysis in FLS, preliminary research in arthritis models has revealed that specific inhibitors of monocarboxylate transporter 4 (MCT4) can effectively restrict the cellular efflux of lactate and protons (H+) (89). Assuming this mechanism is applicable to gout, blocking MCT4 shows potential for fundamentally correcting the pathological acidification of the synovial microenvironment. This intervention would, in turn, delay or prevent the thermodynamic supersaturation and subsequent precipitation of urate crystals.

### 4.4 Exploring the multi-targeted therapeutic potential of traditional Chinese Medicine monomers and formulas

In translational clinical practice, natural Traditional Chinese Medicine (TCM) monomers and classical formulas have demonstrated exceptional multi-targeted efficacy in the long-term management of gout. To integrate these traditional therapeutics into modern precision medicine intervention strategies, it is imperative to rigorously delineate their established pharmacological facts. Building upon this foundation, we must explore their potential mechanistic overlap with the “dual carrier-dual cell” coupling hypothesis proposed herein.

#### 4.4.1 Therapeutic exploration of natural TCM monomers

Current basic research provides robust qualitative and quantitative evidence for the multidimensional mechanisms of TCM monomers in gout therapy. For instance, Berberine (BBR) has been proven to significantly suppress MSU-induced NET formation. *In vivo* studies utilizing DNA laddering and diphenylamine (DPA) assays demonstrated that BBR administration drastically reduces DNA fragmentation rates, concurrently downregulating the expression of neutrophil elastase and myeloperoxidase (90, 91). It is increasingly recognized that the massive vesiculation and shedding of highly pathogenic outer membrane vesicles (OMVs) by pathobionts (such as *Prevotella* and Escherichia coli) are heavily triggered by the severe luminal oxidative stress (ROS) characteristic of the HUA gut. Given the potent ROS-scavenging properties of lipophilic alkaloids like BBR, we cautiously hypothesize that by neutralizing the oxidative microenvironment within the intestinal lumen, BBR might alleviate environmental stress on pathobionts, thereby potentially reducing stress-induced envelope dissolution and OMV blebbing. However, direct experimental evidence linking TCM monomers to the dynamic regulation of bacterial OMV secretion is currently lacking. Future biophysical validations utilizing Nanoparticle Tracking Analysis (NTA) in *in vitro* bacterial cultures are urgently required to empirically test this hypothesis. Similarly, isoquinoline alkaloids such as Palmatine (PAL)—a core active component of Phellodendron amurense—exhibit potent anti-inflammatory activities (57). Both *in vitro* and *in vivo* models confirm that PAL directly blocks MSU-induced NLRP3 inflammasome assembly (inhibiting Caspase-1 and IL-1*β* maturation) by suppressing NF-*κ*B phosphorylation, while concurrently activating the Nrf2/HO-1 pathway to significantly reduce local oxidative stress (ROS) and lipid peroxidation (MDA) levels. Integrating these findings into our “Metabolic-Epigenetic Coupling” framework, we hypothesize that PAL’s robust ROS-scavenging capacity might interfere with the glycolytic acidification of FLS or alter the ratio of key metabolic intermediates (e.g., succinate/*α*-ketoglutarate) in macrophages. Such metabolic rewiring could indirectly inhibit METTL3 activity or activate demethylases (FTO/ALKBH5), thereby erasing the pro-inflammatory m^6^A imprints on pro-IL-1*β* transcripts at the post-transcriptional level. To validate this theoretical epigenetic erasure, future studies must integrate m^6^A sequencing (MeRIP-seq) with metabolic flux analysis to precisely evaluate how TCM monomers remodel the m^6^A methylation landscape of synovial macrophages under OMV stimulation.

#### 4.4.2 Therapeutic exploration of classical TCM formulas

Building upon the mechanisms of single monomers, classical formulas represented by Simiao Decoction (SMD, whose core herb pair includes Atractylodes lancea and Phellodendron amurense) exhibit more macroscopic, system-level regulatory capabilities. Recent studies based on 16S rRNA sequencing and multi-omics confirm that, alongside significantly reducing serum uric acid and local inflammation, the core mechanism of SMD lies in the profound remodeling of the gut microecology and local immunity (92). SMD has been proven to specifically and significantly reduce the abundance of potential pathological bacteria, such as *Prevotella*, in the gut of gouty mice. Furthermore, SMD markedly downregulates local intestinal NLRP3 inflammasome activation and related pro-inflammatory cytokines (e.g., TNF-*α*, IL1*β*), inhibits intestinal apoptotic pathways, and broadly regulates the host’s lipid metabolism network. Integrating our previous discussion on the active secretion of highly pathogenic OMVs by *Prevotella* in gout, we postulate that the multi-targeted network effects of SMD physically sever the source supply of specific OMVs generated under HUA conditions. Simultaneously, SMD’s profound inhibition of local intestinal apoptosis and NLRP3 activation likely structurally mitigates the physical disintegration of the intestinal barrier under inflammatory stress, thereby reducing the passive leakage of free LPS. We hypothesize that this source-level blockade of systemic “dual-carrier” delivery protects distal synovial cells from continuous, high-intensity immunological priming, potentially dismantling the material basis for recurrent gout flares. Future research designs should transcend simplistic gut microbiota abundance sequencing. Fluorescently labeled bacterial OMV *in vivo* tracking technologies could be employed to visually determine whether SMD treatment significantly blocks the cross-organ translocation of gut-derived OMVs to the articular synovium in living organisms. Concurrently, it is necessary to confirm whether the anti-inflammatory and anti-apoptotic effects of SMD truly translate into the substantial restoration of intestinal barrier physical permeability. This would provide the most direct biophysical evidence for classical TCM formulas, severing the “gut-joint” communication.

## 5 Hypotheses and future directions

The progression of gout from asymptomatic HUA to acute flares and chronic persistence constitutes a complex systemic network driven by multidimensional cellular cross-talk. Based on the critical integration of “gut-joint axis” pathogenic communication, cellular metabolic reprogramming, and epitranscriptomics, we formally propose a speculative ‘Two-Hit’ theoretical framework mediated by OMVs and m^6^A Epigenetics.” This aims to provide a hypothetical yet unified conceptual framework for mechanistic breakthroughs and targeted therapies in this field. The core tenet of this hypothesis posits that the pathogenesis of gout requires the convergence of two distinct spatial dysfunctions: a ‘Leaky Gut’ and a ‘Leaky Joint’. Initially, pre-existing biomechanical wear and subclinical osteoarthritis establish a structurally compromised and uric acid-supersaturated microenvironment in the distal joints, providing a baseline vulnerability. Concurrently, intestinal dysbiosis under a HUA background facilitates the precise delivery of specific pathogenic carriers—primarily OMVs and LPS—into the systemic circulation. These gut-derived carriers home in on the structurally compromised joints to execute a spatially and temporally coordinated ‘Two-Hit’ strike. Specifically, LPS derived from pathogenic bacteria—translocating into the bloodstream via a “leaky gut” under HUA conditions—targets SMs. By activating METTL3-mediated m^6^A methylation, this process drastically enhances the stability of NLRP3 and pro-IL-1*β* transcripts at the post-transcriptional level, thereby preemptively establishing a high-intensity immunological priming within macrophages. Synchronously, intestinal-derived OMVs target synovial FLS, inducing intense aerobic glycolysis. The resulting sustained acidification of the local microenvironment, superimposed with the characteristic lower physical temperatures of distal joints, thermodynamically forces the *in situ* precipitation of MSU crystals. This precipitation supplies the requisite physical trigger to fully activate the primed macrophages. Together, these events constitute the underlying molecular logic governing the acute flare of gout and its subsequent transition into chronic inflammatory memory. To advance the scientific rigor within this field, it is imperative to establish strict academic boundaries and critically distinguish between established experimental evidence and theoretical extrapolation. While the individual paradigms of PAMP-induced macrophage m^6^A modification and MSU crystal-driven inflammation are well-documented in adjacent inflammatory models, the continuous theoretical coupling proposed herein—specifically the link between “macrophage m^6^A epigenetic memory” and “OMV-driven FLS metabolic collapse” during the pathogenesis of human gout—currently lacks direct *in vivo* validation in specific gout models or patient-derived clinical cohorts. Consequently, it must be acknowledged that this conceptual “gut-joint” framework is partially founded upon cross-disease extrapolations, the pathways described should be interpreted as a predictive model rather than established clinical facts. Its core value, therefore, lies not in presenting a finalized pathology but rather in providing a heuristic, mechanistic blueprint to guide and stimulate future targeted investigations.

To validate, enrich, and translate this conceptual framework into clinical interventions, future research must explore several macroscopic dimensions. Investigations must transcend simplistic “microbial abundance” analyses and pivot toward exploring how the specific HUA microenvironment in the gut drives the adaptive evolution of pathogenic bacteria. Emphasis should be placed on how this structural variation endows OMVs with the capacity to evade systemic clearance and specifically traffic to distal synovial tissues. Despite the theoretical elegance of this dual-carrier delivery system, a critical pharmacokinetic paradox must be rigorously addressed: how do intestinal OMVs and LPS-laden host EVs, after undergoing exponential dilution in the systemic circulation, achieve the threshold concentration required to activate the CD14/TLR4METTL3 cascade in distal synovial cells? We postulate that this is not achieved through a single acute spike, but rather via chronic spatiotemporal accumulation. Anatomically, the semi-closed physical space and unique microvasculature of the synovial cavity may trap these nanoscale vesicles, allowing their localized concentration to progressively build up over time. Furthermore, the HUA microenvironment itself, combined with the synergistic effects of various gut-derived PAMPs, may drastically lower the basal activation threshold of synovial macrophages. Nevertheless, the absolute quantification of trans-organ delivery efficiency remains a major knowledge gap. Future investigations deploying high-resolution *in vivo* vesicle tracking technologies and quantitative synovial lipidomics are urgently required to empirically determine whether the localized payload of these dual-vesicle carriers naturally breaches the epigenetic activation threshold *in vivo*. Concurrently, traditional *in vitro* single-cell models fail to replicate the authentic dynamic processes of gout flares. Future mechanistic explorations must leverage high-dimensional, high-resolution spatial multi-omics technologies to decode the microenvironmental network at the *in vivo* joint level. Key imperatives include elucidating the spatial matching mechanisms between the metabolic heterogeneity of FLS and the *in situ* crystallization kinetics of MSU, as well as determining whether the m^6^A methylation landscape of synovial macrophages undergoes dynamic erasure and rewriting along the disease continuum.

Based on this “OMVs-m^6^A-mediated Two-Hit model within the gut-joint axis,” future clinical management and drug development pipelines must undergo a paradigm shift. On one front, exploring “circulatory interception” technologies based on biomimetic nanomaterials, or discovering natural product molecules capable of interfering with bacterial envelope integrity, will provide novel avenues to sever gut-joint communication. Conversely, addressing the localized chronic inflammatory memory within the synovium by developing small-molecule modulators that precisely target specific metabolic enzymes or specifically intervene in the activity of core m^6^A modifying enzymes represents a highly promising direction for breaking the cycle of disease recurrence. However, it must be acknowledged that several of these targeted strategies remain at the proof-of-concept stage. Future clinical translation faces formidable challenges: relying on large-scale prospective cohorts, the clinical potential of the absolute circulating OMV payload and the m^6^A modification levels in peripheral immune cells as biomarkers for the “precision subtyping” of gout must be rigorously mined. For emerging therapies and TCM multi-target interventions, systemic off-target effects and long-term pharmacokinetic profiles must be stringently evaluated. Ultimately, only through high-quality randomized controlled trials (RCTs) can the long-term safety and clinical potential of these systemic intervention strategies be comprehensively evaluated.

## References

[1] Tian P Zhao H Wang B Chen Y Jia Z Wang C . Global burden of gout among young people from 1990 to 2021, with projections for 2050: A systematic analysis based on the global burden of disease study 2021. PloS One. (2025) 20:e0333368. doi: 10.1371/journal.pone.0333368 41160620 PMC12571247

[2] Afinogenova Y Danve A Neogi T . Update on gout management: what is old and what is new. Curr Opin Rheumatol. (2022) 34:118–24. doi: 10.1097/bor.0000000000000861 34907116 PMC8799507

[3] Galozzi P Bindoli S Doria A Oliviero F Sfriso P . Autoinflammatory features in gouty arthritis. J Clin Med. (2021) 10:1880. doi: 10.3390/jcm10091880 33926105 PMC8123608

[4] Xu X Wang M Wang Z Chen Q Chen X Xu Y . The bridge of the gut–joint axis: Gut microbial metabolites in rheumatoid arthritis. Front Immunol. (2022) 13:1007610. doi: 10.3389/fimmu.2022.1007610 36275747 PMC9583880

[5] Chen P Luo Z Lu C Jian G Qi X Xiong H . Gut-immunity-joint axis: a new therapeutic target for gouty arthritis. Front Pharmacol. (2024) 15:1353615. doi: 10.3389/fphar.2024.1353615 38464719 PMC10920255

[6] Jan AT . Outer membrane vesicles (omvs) of gram-negative bacteria: a perspective update. Front Microbiol. (2017) 8:1053. doi: 10.3389/fmicb.2017.01053 28649237 PMC5465292

[7] Chu Y Sun S Huang Y Gao Q Xie X Wang P . Metagenomic analysis revealed the potential role of gut microbiome in gout. NPJ Biofilms Microbiomes. (2021) 7:66. doi: 10.1038/s41522-021-00235-2 34373464 PMC8352958

[8] Sartorio MG Pardue EJ Feldman MF Haurat MF . Bacterial outer membrane vesicles: From discovery to applications. Annu Rev Microbiol. (2021) 75:609–30. doi: 10.1146/annurev-micro-052821-031444 34351789 PMC8500939

[9] Lu J Wang Y Wu J Duan Y Zhang H . Linking microbial communities to rheumatoid arthritis: Focus on gut, oral microbiome and their extracellular vesicles. Front Immunol. (2025) 16:1503474. doi: 10.3389/fimmu.2025.1503474 40308573 PMC12040682

[10] Su W Lu Y Luo Z . Epigenetic programming reshapes innate immune memory: Decoding the molecular imprint of gouty inflammation. Front Pharmacol. (2025) 16:1678958. doi: 10.3389/fphar.2025.1678958 41311848 PMC12647090

[11] Guo Z Zhang J Wang Z Ang KY Huang S Hou Q . Intestinal microbiota distinguish gout patients from healthy humans. Sci Rep. (2016) 6:20602. doi: 10.1038/srep20602 26852926 PMC4757479

[12] Yang X Liu D Zhao X Han Y Zhang X Zhou Q . Hyperuricemia drives intestinal barrier dysfunction by regulating gut microbiota. Heliyon. (2024) 10:e36024. doi: 10.1016/j.heliyon.2024.e36024 39224259 PMC11367111

[13] Qie J Cao M Xu M Zhang Y Luo L Sun C . Multi-cohort analysis unveils novel microbial targets for the treatment of hyperuricemia and gout. Msystems. (2025) 10:e01091–25. doi: 10.1128/msystems.01091-25 40960303 PMC12542679

[14] Danve A Sehra ST Neogi T . Role of diet in hyperuricemia and gout. Best Pract Res Clin Rheumatol. (2021) 35:101723. doi: 10.1016/j.berh.2021.101723 34802900 PMC8678356

[15] Punzi L Scagnellato L Galozzi P Baggio C Damasco A Oliviero F . Gout: One year in review 2025. Clin Exp Rheumatol. (2025) 43:799–808. doi: 10.55563/clinexprheumatol/9sdln5 40153315

[16] Emery HL Kerby RL Rey FE . The central role of gut microbes in host purine homeostasis. Annu Rev Microbiol. (2025) 79:615–38. doi: 10.1146/annurev-micro-041522-100126 40902203

[17] Liu Y Jarman JB Low YS Augustijn HE Huang S Chen H . A widely distributed gene cluster compensates for uricase loss in hominids. Cell. (2023) 186:3400–13. doi: 10.1016/j.cell.2023.06.010 37541197 PMC10421625

[18] Liu Y Zhou Z Jarman JB Chen H Miranda-Velez M Terkeltaub R . Gut bacteria degrade purines via the 2, 8-dioxopurine pathway. Nat Microbiol. (2025) 10:2291–305. doi: 10.1038/s41564-025-02079-4 40770490 PMC12666987

[19] Fang X Qi L Chen H Gao P Zhang Q Leng R . The interaction between dietary fructose and gut microbiota in hyperuricemia and gout. Front Nutr. (2022) 9:890730. doi: 10.3389/fnut.2022.890730 35811965 PMC9257186

[20] Liu X Feng Z Zhang F Wang B Wei Z Liao N . Causal effects of gut microbiota on gout and hyperuricemia: Insights from genome-wide mendelian randomization, rna-sequencing, 16s rrna sequencing, and metabolomes. Biosci Rep. (2024) 44:BSR20240595. doi: 10.1042/bsr20240595 39492788 PMC11598824

[21] Major TJ Takei R Matsuo H Leask MP Sumpter NA Topless RK . A genome-wide association analysis reveals new pathogenic pathways in gout. Nat Genet. (2024) 56:2392–406. doi: 10.1038/s41588-024-01921-5 39406924

[22] Chen G Ran X Li B Li Y He D Huang B . Sodium butyrate inhibits inflammation and maintains epithelium barrier integrity in a tnbs-induced inflammatory bowel disease mice model. EBioMedicine. (2018) 30:317–25. doi: 10.1016/j.ebiom.2018.03.030 29627390 PMC5952406

[23] Martín R Rios-Covian D Huillet E Auger S Khazaal S Bermúdez-Humarán LG . Faecalibacterium: A bacterial genus with promising human health applications. FEMS Microbiol Rev. (2023) 47:fuad039. doi: 10.1093/femsre/fuad039 37451743 PMC10410495

[24] Vatanen T Kostic AD d’Hennezel E Siljander H Franzosa EA Yassour M . Variation in microbiome lps immunogenicity contributes to autoimmunity in humans. Cell. (2016) 165:842–53. doi: 10.1016/j.cell.2016.04.007 27133167 PMC4950857

[25] Shin JH Tillotson G MacKenzie TN Warren CA Wexler HM . Bacteroides and related species: The keystone taxa of the human gut microbiota. Anaerobe. (2024) 85:102819. doi: 10.1016/j.anaerobe.2024.102819 38215933

[26] Cani PD Bibiloni R Knauf C Waget A Neyrinck AM Delzenne NM . Changes in gut microbiota control metabolic endotoxemia-induced inflammation in high-fat diet–induced obesity and diabetes in mice. Diabetes. (2008) 57:1470–81. doi: 10.2337/db07-1403 18305141

[27] Stoll ML . Genetics, prevotella, and the pathogenesis of rheumatoid arthritis. Lancet Rheumatol. (2020) 2:e375–6. doi: 10.1016/s2665-9913(20)30090-4 38273603

[28] Segev-Zarko L Kapach G Josten M Klug YA Sahl H-G . Deficient lipid a remodeling by the arnb gene promotes biofilm formation in antimicrobial peptide susceptible pseudomonas aeruginosa. Biochemistry. (2018) 57:2024–34. doi: 10.1021/acs.biochem.8b00149 29518324

[29] Thaipisuttikul I Hittle LE Chandra R Zangari D Dixon CL Garrett TA . A divergent p seudomonas aeruginosa palmitoyltransferase essential for cystic fibrosis-specific lipid a. Mol Microbiol. (2014) 91:158–74. 10.1111/mmi.12451 PMC3935289 24283944

[30] Schultz KM Schneider JR Fischer MA Cina NP Riegert MO Frank DW . Binding and transport of lps occurs through the coordinated combination of an array of sites across the entire escherichia coli lps transport protein lpta. Protein Sci. (2023) 32:e4724. doi: 10.1002/pro.4724 37417889 PMC10360375

[31] Crane JK Broome JE Lis A . Biological activities of uric acid in infection due to enteropathogenic and shiga-toxigenic escherichia coli. Infect Immun. (2016) 84:976–88. doi: 10.1128/iai.01389-15 26787720 PMC4807499

[32] Christovich A Luo XM . Gut microbiota, leaky gut, and autoimmune diseases. Front Immunol. (2022) 13:946248. doi: 10.3389/fimmu.2022.946248 35833129 PMC9271567

[33] Wang Y Li L Chen S Yu Z Gao X Peng X . Faecalibacterium prausnitziiderived extracellular vesicles alleviate chronic colitis-related intestinal fibrosis by macrophage metabolic reprogramming. Pharmacol Res. (2024) 206:107277. doi: 10.1016/j.phrs.2024.107277 38945379

[34] Zafar H . Gut bacteroides species in health and disease. Gut Microbes. (2021) 13:1848158. doi: 10.1080/19490976.2020.1848158 33535896 PMC7872030

[35] Fujita K Ichida K . Abcg2 as a therapeutic target candidate for gout. Expert Opin Ther Targets. (2018) 22:123–9. doi: 10.1080/14728222.2018.1420167 29264928

[36] Han R Wang Z Li Y Ke L Li X Li C . Gut microbiota lactobacillus johnsonii alleviates hyperuricemia by modulating intestinal urate and gut microbiota-derived butyrate. Chin Med J. (2026) 139:118–35. doi: 10.1097/cm9.0000000000003603 40304365 PMC12767932

[37] Terkeltaub R . The gut microbiome in hyperuricemia and gout. Arthritis Rheumatol. (2025) 77:955–65. doi: 10.1002/art.43118 39829115 PMC12276925

[38] Wang M Fan J Huang Z Zhou D . Causal relationship between gut microbiota and gout: A two-sample mendelian randomization study. Nutrients. (2023) 15:4260. doi: 10.3390/nu15194260 37836544 PMC10574468

[39] Scher JU Sczesnak A Longman RS Segata N Ubeda C Bielski C . Expansion of intestinal prevotella copri correlates with enhanced susceptibility to arthritis. Elife. (2013) 2:e01202. doi: 10.7554/elife.01202 24192039 PMC3816614

[40] Kasahara K Kerby RL Zhang Q Pradhan M Mehrabian M Lusis AJ . Gut bacterial metabolism contributes to host global purine homeostasis. Cell Host Microbe. (2023) 31:1038–53. doi: 10.1016/j.chom.2023.05.011 37279756 PMC10311284

[41] Lu J Liu G Sun W Jia G Zhao H Chen X . Dietary α-ketoglutarate alleviates escherichia coli lps-induced intestinal barrier injury by modulating the endoplasmic reticulum-mitochondrial system pathway in piglets. J Nutr. (2024) 154:2087–96. doi: 10.1016/j.tjnut.2024.03.001 38453028

[42] Bagaev AV Garaeva AY Lebedeva ES Pichugin AV Ataullakhanov RI Ataullakhanov FI . Elevated pre-activation basal level of nuclear nf-κB in native macrophages accelerates lps-induced translocation of cytosolic nf-κB into the cell nucleus. Sci Rep. (2019) 9:4563. doi: 10.1038/s41598-018-36052-5 30872589 PMC6418260

[43] Ryu J-K Kim SJ Rah S-H Kang JI Jung HE Lee D . Reconstruction of lps transfer cascade reveals structural determinants within lbp, cd14, and tlr4-md2 for efficient lps recognition and transfer. Immunity. (2017) 46:38–50. doi: 10.1016/j.immuni.2016.11.007 27986454

[44] Thery C Witwer KW Aikawa E Alcaraz MJ Anderson JD Andriantsitohaina R . Minimal information for studies of extracellular vesicles 2018 (misev2018): A position statement of the international society for extracellular vesicles and update of the misev2014 guidelines. J Extracell Vesicles. (2018) 7:1535750. doi: 10.1080/20013078.2018.1535750 30637094 PMC6322352

[45] Kumari P Vasudevan SO Russo AJ Wright SS Fraile-Agreda V Krajewski D . Host extracellular vesicles confer cytosolic access to systemic lps licensing non-canonical inflammasome sensing and pyroptosis. Nat Cell Biol. (2023) 25:1860–72. doi: 10.1038/s41556-023-01269-8 37973841 PMC11111309

[46] Huang Y Nieh M-P Chen W Lei Y . Outer membrane vesicles (omvs) enabled bio-applications: a critical review. Biotechnol Bioeng. (2022) 119:34–47. doi: 10.1002/bit.27965 34698385

[47] Chen S Lei Q Zou X Ma D . The role and mechanisms of gram-negative bacterial outer membrane vesicles in inflammatory diseases. Front Immunol. (2023) 14:1157813. doi: 10.3389/fimmu.2023.1157813 37398647 PMC10313905

[48] Kaparakis-Liaskos M Ferrero RL . Immune modulation by bacterial outer membrane vesicles. Nat Rev Immunol. (2015) 15:375–87. doi: 10.1038/nri3837 25976515

[49] Maeda Y Rabenow M Xiang W Schmid E Otterbein N Gimaev I . Mucosal innate immune activation as the trigger to prevotella species-induced arthritis in genetically resistant mice. Cell Rep. (2026) 45:116755. doi: 10.1016/j.celrep.2025.116755 41499240

[50] Li M Zhou H Yang C Wu Y Zhou X Liu H . Bacterial outer membrane vesicles as a platform for biomedical applications: an update. J Controlled Rel. (2020) 323:253–68. doi: 10.1016/j.jconrel.2020.04.031 32333919

[51] Vanaja SK Russo AJ Behl B Banerjee I Yankova M Deshmukh SD . Bacterial outer membrane vesicles mediate cytosolic localization of lps and caspase-11 activation. Cell. (2016) 165:1106–19. doi: 10.1016/j.cell.2016.04.015 27156449 PMC4874922

[52] Desai J Steiger S Anders H-J . Molecular pathophysiology of gout. Trends Mol Med. (2017) 23:756–68. doi: 10.1016/j.molmed.2017.06.005 28732688

[53] Sun L Chen X Zhu S Wang J Diao S Liu J . Decoding m^6^A mrna methylation by reader proteins in liver diseases. Genes Dis. (2024) 11:711–26. doi: 10.1016/j.gendis.2023.02.054 PMC10491919 37692496

[54] Wang J Wang F Ke J Li Z Xu C Yang Q . Inhibition of mettl3 attenuates renal injury and inflammation by alleviating tab3 m^6^A modifications via igf2bp2-dependent mechanisms. Sci Transl Med. (2022) 14:eabk2709. doi: 10.1126/scitranslmed.abk2709 35417191

[55] Fu Y Dominissini D Rechavi G He C . Gene expression regulation mediated through reversible m^6^A rna methylation. Nat Rev Genet. (2014) 15:293–306. doi: 10.1038/nrg3724 24662220

[56] Shi Q Li Z Dong Y Yang G . Lncrna thril, transcriptionally activated by ap-1 and stabilized by mettl14-mediated m^6^A modification, accelerates lps-evoked acute injury in alveolar epithelial cells. Int Immunopharmacol. (2023) 123:110740. doi: 10.1016/j.intimp.2023.110740 37543013

[57] Cheng J-J Ma X-D Ai G-X Yu Q-X Chen X-Y Yan F . Palmatine protects against msu-induced gouty arthritis via regulating the nf-κB/nlrp3 and nrf2 pathways. Drug Des Dev Ther. (2022) 16:2119–32. doi: 10.2147/dddt.s356307 35812134 PMC9259749

[58] Fu J Zong X Zhang H Zhu L Gong T Cheng Y . Mettl3-mediated m^6^A on nascent rna coordinates translational and transcriptional programs to activate the nlrp3 inflammasome in macrophages. Cell Rep. (2026) 45:116808. doi: 10.1016/j.celrep.2025.116808 41520337

[59] Aghakhani S Soliman S Niarakis A . Metabolic reprogramming in rheumatoid arthritis synovial fibroblasts: a hybrid modeling approach. PloS Comput Biol. (2022) 18:e1010408. doi: 10.1371/journal.pcbi.1010408 36508473 PMC9779668

[60] Chen X Zhang C Zheng H Shi Q Chen B Han J . Gout inflammation time programming: molecular clock from crystal triggering to tissue remodeling. Int J Mol Sci. (2026) 27:1523. doi: 10.3390/ijms27031523 41683942 PMC12897657

[61] FitzGerald O Bresnihan B . Synovial membrane cellularity and vascularity. Ann Rheumatic Dis. (1995) 54:511. doi: 10.1136/ard.54.6.511 7632098 PMC1009914

[62] Cehakova M Ivanisova D Strecanska M Plava J Varchulova Novakova Z Nicodemou A . Rheumatoid synovial fluid and acidic extracellular ph modulate the immunomodulatory activity of urine-derived stem cells. Int J Mol Sci. (2023) 24:15856. doi: 10.3390/ijms242115856 37958839 PMC10648750

[63] Narang RK Dalbeth N . Pathophysiology of gout. Semin Nephrol. (2020) 40:550–63 doi: 10.1016/j.semnephrol.2020.12.001 33678310

[64] Zhou Y Song K Tu B Sun H Ding J-F Luo Y . Mettl3 boosts glycolysis and cardiac fibroblast proliferation by increasing ar methylation. Int J Biol Macromol. (2022) 223:899–915. doi: 10.1016/j.ijbiomac.2022.11.042 36370857

[65] Li X Ling M Wen Z Jiang C Tan X . Usp5 promotes glycolysis of fibroblast-like synoviocytes by stabilizing the mettl14/m^6^A/glut1 axis in rheumatoid arthritis. Cell Death Discov. (2025) 12:32. doi: 10.1038/s41420-025-02890-2 41339332 PMC12811265

[66] Tang R Qin S Xu Q Lin W Zhang S Peng Y . Lipopolysaccharide-induced histone lactylation mediates m^6^A rna modification causing mitochondrial dysfunction and pulmonary fibroblasts activation to exacerbate sepsis-associated pulmonary fibrosis. Respir Res. (2025) 26:347. doi: 10.1186/s12931-025-03422-3 41402767 PMC12709686

[67] Gout. Ann Internal Med. (2025) 178:ITC33–48. doi: 10.7326/ANNALS-24-03951 40063960

[68] Mitroulis I Kambas K Chrysanthopoulou A Skendros P Apostolidou E Kourtzelis I . Neutrophil extracellular trap formation is associated with il-1β and autophagy-related signaling in gout. PloS One. (2011) 6:e29318. doi: 10.1371/journal.pone.0029318 22195044 PMC3241704

[69] Wang K Li J Li J Zeng F Li S Chen P . Spatiotemporal immune gradients in gout: Immune response–driven activation of the nlrp3–il-1β axis and its transition to trained immunity. Front Immunol. (2026) 17:1776479. doi: 10.3389/fimmu.2026.1776479 41836392 PMC12982045

[70] Schauer C Janko C Munoz LE Zhao Y Kienhofer D Frey B . Aggregated neutrophil extracellular traps limit inflammation by degrading cytokines and chemokines. Nat Med. (2014) 20:511–7. doi: 10.1038/nm.3547 24784231

[71] Jing C Castro-Dopico T Richoz N Tuong ZK Ferdinand JR Lok LS . Macrophage metabolic reprogramming presents a therapeutic target in lupus nephritis. Proc Natl Acad Sci. (2020) 117:15160–71. doi: 10.1073/pnas.2000943117 32541026 PMC7334513

[72] Chen Y Zhao L Li J Li H Zhang N . Harnessing nanotechnology for gout therapy: colchicine-loaded nanoparticles regulate macrophage polarization and reduce inflammation. Biomater Res. (2024) 28:89. doi: 10.34133/bmr.0089 39665079 PMC11632155

[73] Zhu S Jiang L Liu X Chen C Luo X Jiang S . m^6^A demethylase fto inhibited macrophage activation and glycolysis in diabetic nephropathy via m^6^A/npas2/HIF-1α axis. FASEB J. (2025) 39:e70332. doi: 10.1096/fj.202403014R 39831513 PMC11744739

[74] Greer EL Shi Y . Histone methylation: a dynamic mark in health, disease and inheritance. Nat Rev Genet. (2012) 13:343–57. doi: 10.1038/nrg3173 22473383 PMC4073795

[75] Arts RJ Novakovic B Ter Horst R Carvalho A Bekkering S Lachmandas E . Glutaminolysis and fumarate accumulation integrate immunometabolic and epigenetic programs in trained immunity. Cell Metab. (2016) 24:807–19. doi: 10.1016/j.cmet.2016.10.008 27866838 PMC5742541

[76] Pieterse E Jeremic I Czegley C Weidner D Biermann MH Veissi S . Bloodborne phagocytes internalize urate microaggregates and prevent intravascular netosis by urate crystals. Sci Rep. (2016) 6:38229. doi: 10.1038/srep38229 27917897 PMC5137018

[77] Chhana A Dalbeth N . The gouty tophus: a review. Curr Rheumatol Rep. (2015) 17:19. doi: 10.1007/s11926-014-0492-x 25761926

[78] Liu Q Gu W Ma J Wang J Yu M Xu M . A pilot randomized controlled trial and multi-omics analysis of electrolysed alkaline water: Impacts on gut microbiota and metabolic signatures in hyperuricemia. Nutrients. (2025) 18:107. doi: 10.3390/nu18010107 41515223 PMC12787813

[79] Gencer G Mancuso C Chua KJ Ling H Costello CM Chang MW . Engineering escherichia coli for diagnosis and management of hyperuricemia. Front Bioeng Biotechnol. (2023) 11:1191162. doi: 10.3389/fbioe.2023.1191162 37288353 PMC10242094

[80] Amalia L Tsai S-L . Functionalization of omvs for biocatalytic applications. Membranes. (2023) 13:459. doi: 10.3390/membranes13050459 37233521 PMC10222932

[81] Batista JH Leal FC Fukuda TT Alcoforado Diniz J Almeida F Pupo MT . Interplay between two quorum sensing-regulated pathways, violacein biosynthesis and vacj/yrb, dictates outer membrane vesicle biogenesis in chromobacterium violaceum. Environ Microbiol. (2020) 22:2432–42. doi: 10.1111/1462-2920.15033 32329144

[82] Huang X Zhang W . Macrophage membrane-camouflaged biomimetic nanovesicles for targeted treatment of arthritis. Ageing Res Rev. (2024) 95:102241. doi: 10.1016/j.arr.2024.102241 38387516

[83] Liu Y Rao P Qian H Shi Y Chen S Lan J . Regulatory fibroblast-like synoviocytes cell membrane coated nanoparticles: A novel targeted therapy for rheumatoid arthritis. Adv Sci. (2023) 10:2204998. doi: 10.1002/advs.202204998 36509660 PMC9896074

[84] Zhao X Wei Y Bu Y Ren X . Review on bacterial outer membrane vesicles: Structure, vesicle formation, separation and biotechnological applications. Microb Cell Fact. (2025) 24:27. doi: 10.1186/s12934-025-02653-9 39833809 PMC11749425

[85] Lv X Zhang W Liu Y Hu Y Wang X . Epigenetic modifier m^6^A methylation: Insights into the pathogenesis and therapeutic potential of autoimmune diseases. J Transl Med. (2025) 23:1343. doi: 10.1186/s12967-025-07347-9 41286919 PMC12642297

[86] He M Liu J Sun Y Fang Y Wang F . Novel insights into the regulatory role of n^6^-methyladenosine in the pathogenesis and clinical treatment of osteoarthritis: research status and prospect. J Inflammation Res. (2025) 18:6749–66. doi: 10.2147/jir.s508973 40453975 PMC12126110

[87] Li R Kuang Y Niu Y Zhang S Chen S Su F . Fto-mediated rna m^6^A methylation regulates synovial aggression and inflammation in rheumatoid arthritis. Biochim Biophys Acta (BBA)-Molecular Basis Dis. (2024) 1870:167341. doi: 10.1016/j.bbadis.2024.167341 39025373

[88] Cui L-G Wang S-H Komal S Yin J-J Zhai M-M Zhou Y-J . Alkbh5 promotes cardiac fibroblasts pyroptosis after myocardial infarction through notch1/nlrp3 pathway. Cell Signall. (2025) 127:111574. doi: 10.1016/j.cellsig.2024.111574 39710090

[89] Pucino V Certo M Bulusu V Cucchi D Goldmann K Pontarini E . Lactate buildup at the site of chronic inflammation promotes disease by inducing cd4+ t cell metabolic rewiring. Cell Metab. (2019) 30:1055–74. doi: 10.1016/j.cmet.2019.10.004 31708446 PMC6899510

[90] Ibrahim Fouad G Aly HF Shalaby MB Mabrouk MI Khalil WK Rizk MZ . Antiarthritic activities of berberine in a rat model of gouty arthritis. Sci Rep. (2025) 15:32153. doi: 10.1038/s41598-025-16622-0 40890319 PMC12402284

[91] Xu X Yi H Wu J Kuang T Zhang J Li Q . Therapeutic effect of berberine on metabolic diseases: Both pharmacological data and clinical evidence. Biomed Pharmacother. (2021) 133:110984. doi: 10.1016/j.biopha.2020.110984 33186794

[92] Lin X Shao T Huang L Wen X Wang M Wen C . Simiao decoction alleviates gouty arthritis by modulating proinflammatory cytokines and the gut ecosystem. Front Pharmacol. (2020) 11:955. doi: 10.3389/fphar.2020.00955 32670069 PMC7327538

